# Novel Insights and Future Perspective in Iron Metabolism and Anemia

**DOI:** 10.3390/metabo12020138

**Published:** 2022-02-02

**Authors:** Immacolata Andolfo, Roberta Russo

**Affiliations:** 1 Dipartimento di Medicina Molecolare e Biotecnologie Mediche, Università degli Studi di Napoli Federico II, 80131 Napoli, Italy; 2 CEINGE Biotecnologie Avanzate, 80145 Napoli, Italy

Iron is an essential element for nearly all living organisms. In the last 20 years, research in this field has clarified the role of iron in physiology and diseases, providing not only a better definition of the physiological role of iron in mammals but also influencing the therapeutic approaches of iron and iron-related erythroid disorders [1]. Iron is necessary for hemoglobin synthesis and crucial for all cells for the production of heme and iron-sulfur clusters, which are components of the proteins/enzymes involved in vital biological processes, such as respiration, nucleic acid replication and repair, metabolic reactions, and host defense [2]. Iron is indispensable for life, but excess iron is toxic. The ability to accept/release electrons explains the tendency of iron to damage cell components, and it is the reason why body iron must be finely regulated. Iron homeostasis is maintained by regulating intestinal iron absorption, iron concentration in blood plasma and extracellular fluid, its distribution among organs and tissues, and the amount of stored iron [3]. The disorders associated with iron dysmetabolism span from iron deficiency to iron overload, as well as iron maldistribution among tissues in which individual tissues or organs may become iron-deficient or iron-overloaded [3]. These different conditions may arise from genetic alterations that directly impair iron regulation, such as hereditary hemochromatosis [4], atypical inherited microcytic anemias [5], IRIDA [6], or conditions that impact iron regulation indirectly [1].

Systemic iron homeostasis is primarily regulated by the erythron compartment, composed of red blood cells and their precursors in erythropoietic organs. A variety of studies in the last 10 years focused on the regulation of the expression of hepatic hormone hepcidin by the erythroid compartment. In 2014, the main erythroid regulator of hepcidin production was identified: the erythroferrone (ERFE) [7]. This is produced by erythroid cells in response to hemorrhage, hypoxia, or other erythropoietic stimuli, and suppresses the hepatic production of hepcidin, thus mobilizing iron for erythropoiesis. The suppression of hepcidin by ERFE is mediated by interference with paracrine BMP/SMADs signaling that regulates hepcidin transcription in hepatocytes [8]. In anemias with ineffective erythropoiesis, such as thalassemias, congenital dyserythropoietic anemias (CDAs), and myelodysplasia, ERFE is pathologically overproduced and related to abnormal erythropoiesis and acts by inhibiting hepatic signaling of BMP/SMADs [9,10]. Despite numerous studies having been carried out on this topic, the contribution of ERFE overexpression to the clinical manifestations of these anemias is not well understood. Indeed, no clear correlation between ERFE levels and iron balance has been proven so far [11,12]. Of note, a recent study demonstrated that ERFE excess causes iron overload even in the absence of other pathology, and high concentrations of ERFE cause growth delay and hypoplastic kidneys in mice [13], thus expanding the phenotypic spectrum associated with dysregulation of this hormone. Currently, no pathogenic variants in the *ERFE* gene have been associated with human diseases. Nevertheless, specific variants have been involved either in the pathogenesis of myelodysplastic syndrome or in the modulation of the clinical phenotype of CDA type II [12,14].

Another emerging crucial regulator of iron homeostasis is the second receptor of transferrin, TFR2 [4,15]. It is mainly expressed in hepatocytes, where it positively regulates hepcidin expression; in erythroid precursors, where it binds EPO receptor; and in osteoblasts and osteoclasts, where it regulates bone mass and mineralization [16]. Mice with selective inactivation of Tfr2 in the bone marrow showed erythrocytosis [17]. In humans, TFR2 loss-of-function mutations cause hemochromatosis [18]. 

The iron storage compartments are also primarily involved in systemic iron homeostasis. Among the key players involved in these processes, recent evidence highlights a role for PIEZO1 in the regulation of iron metabolism. PIEZO1 is a mechanoreceptor involved in several crucial biological processes [19]. Gain-of-function (GoF) mutations in PIEZO1 cause dehydrated hereditary stomatocytosis (DHS) [20]. Patients with DHS show hyperferritinemia and very low levels of plasma hepcidin [21]. Overexpression and chemical activation in hepatoma cell lines of the R2456H and R2488Q PIEZO1 GoF mutants cause increased Ca^2+^ signal associated with ERK phosphorylation, inhibition of the BMP/SMADs pathway, and decreased expression of *HAMP* gene, encoding hepcidin [22]. PIEZO1 involvement in iron metabolism was further confirmed in constitutive and in macrophage-specific transgenic PIEZO1 GoF mice. By 1 year of age, these mice develop severe hepatic hemosiderosis with elevated serum ferritin and transferrin saturation, accompanied by increased erythrophagocytosis, erythropoiesis, and erythroferrone [23]. Increased serum ferritin and transferrin saturation were also observed in the over-40 age subgroup of African Americans carrying the E756del variant in PIEZO1 [24].

Studies of iron metabolism have identified novel iron regulating genes, proteins, and pathways and revealed the essential role of the hepcidin–ferroportin axis in systemic iron homeostasis. Indeed, the immunophilin FKBP12 is another hepcidin regulator. It binds the BMP receptor ALK2, suppressing the BMP/SMADs pathway activation [25]. 

Another critical compartment in systemic iron homeostasis is represented by the absorptive enterocytes in the duodenum through which iron enters the body. Intestinal iron absorption is activated during increased systemic iron demand, as in iron-deficiency anemia. Recently, the importance of the intestinal HIF2a/ferritinophagy axis in systemic iron homeostasis has been shown. Ferritinophagy is a process that mediates the autophagic breakdown of ferritin and subsequent release of iron and it is regulated by the nuclear receptor co-activator 4 (NCOA4) [26]. NCOA4 also mediated ferritinophagy in macrophages to favor iron release for erythropoiesis, especially in iron deficiency [27]. A recent study demonstrated that NCOA4-mediated intestinal ferritinophagy is integrated into systemic iron demand via HIF2a [28].

We hope that the Special Issue on “Advance in Iron Metabolism and Anemia” will further advance our knowledge on the fascinating world of iron metabolism and its related diseases. As guest editors, we would like to thank all the authors that will contribute to this Special Issue, the peer reviewers, and the Metabolites Editorial Office for their support and contributions. 
